# Effects of hemp-based polyunsaturated fatty acid supplementation on membrane lipid profiles and reproductive performance in Martina Franca jacks

**DOI:** 10.3389/fvets.2025.1553218

**Published:** 2025-04-16

**Authors:** Isa Fusaro, Salvatore Parrillo, Giovanni Buonaiuto, Paraskevi Prasinou, Alessandro Gramenzi, Roberta Bucci, Damiano Cavallini, Alessia Carosi, Augusto Carluccio, Ippolito De Amicis

**Affiliations:** ^1^Department of Veterinary Medicine, University of Teramo, Teramo, Italy; ^2^Department of Veterinary Medical Science, Alma Mater Studiorum, University of Bologna, Bologna, Italy

**Keywords:** semen quality, lipidomic, antioxidants, cannabidiol, industrial hemp, donkey nutrition

## Abstract

This study evaluates the impact of dietary supplementation with hemp-based polyunsaturated fatty acids (PUFAs) on the membrane lipid profiles and reproductive performance of Martina Franca jacks. Over a 90-day period, twelve donkeys were divided into a treatment group receiving hemp oil and a control group on a standard diet. Semen and blood samples were collected and analyzed at multiple time points for lipid composition and reproductive parameters. Results revealed that sperm motility improved significantly, increasing from 92.61% in the control group to 96.63% by Day 60 in the treatment group (*p* = 0.05). Normal sperm morphology also showed a significant enhancement, rising from 96.58% in the control group to 98.85% by Day 60 (*p* = 0.04). Conversely, gel-free semen volume decreased significantly in the treatment group, from 64.17 mL in the control group to 28.20 mL at Day 60 (*p* < 0.0001). Lipidomic analyses indicated an increased proportion of omega-3 PUFAs, such as eicosapentaenoic acid (EPA; 0.08% in the control group to 0.20% by Day 60, *p* < 0.0001), in sperm membranes, alongside a reduction in the peroxidation index (264.11 in the control group to 86.53 in the treatment group, *p* < 0.0001). These changes suggest improved membrane fluidity and oxidative stability. These findings underscore the potential of hemp oil as a dietary supplement to enhance reproductive outcomes in donkeys, with broader implications for livestock management.

## 1 Introduction

In recent decades, increasing consumer awareness of social and environmental issues has driven a growing demand for more sustainable animal products. As a result, the livestock sector, has adopted more efficient resource management strategies, particularly regarding soil and water. Among the various approaches explored to reduce the environmental impact of animal production, the inclusion of sustainable feed ingredients has gained considerable interest (1, 2). One crop that has received attention for its sustainability potential is hemp [*Cannabis sativa*; (3)].

Hemp is an annual angiosperm belonging to the Cannabaceae family (4). While the terms “hemp” and “cannabis” are often used interchangeably; “hemp” generally refers to the plant when cultivated for industrial, textile, or culinary purposes (5). In contrast, “cannabis” is commonly associated with the plant’s therapeutic or psychoactive properties (6). Currently, hemp cultivation falls into two distinct categories (7). The first is industrial hemp, characterized by a tetrahydrocannabinol (THC) content of less than 0.2% (8). The second is medical cannabis, which is cultivated under strict quality standards for therapeutic use. Hemp has a wide range of domestic and industrial applications, as extensively documented in previous reviews [e.g., (9–11)]. In medicine, cannabis is employed for its therapeutic potential in treating various conditions in both humans and animals (12–14). According to the European Food Safety Authority (EFSA), hemp seeds and hempseed meal can be included in the diets of various animal species, though the recommended inclusion rates vary depending on species-specific metabolic needs (15). Hemp oil serves as a dietary supplement due to its high EFA content. Research on hemp supplementation in livestock has demonstrated beneficial effects on both animal health and productivity (16–19), yet studies specifically investigating equids remain scarce. Notably, the limited research available has focused primarily on horses, often examining the plant’s pharmaceutical applications (20, 21) or its use as a bedding material (22) rather than as a nutritional supplement.

Equids’ diets are traditionally based on forages, often supplemented with compound feedstuffs (23, 24). Certain equine categories, including those used for meat production, intense physical work (e.g., racehorses, draft donkeys), or breeding, require high-energy diets rich in grains (25, 26). However, excessive starch intake has been linked to metabolic disorders such as gastrointestinal disturbances (27, 28) and laminitis (29), which can compromise both performance and welfare. To mitigate these risks, lipid supplementation has emerged as a viable alternative energy source (30). Among the fat sources used in equine nutrition, vegetable oils have gained increasing due to their positive effects on diet palatability, general health, and animal welfare. Rich in PUFAs and precursors of the omega-3 and omega-6 series, vegetable oils contribute to improved metabolic efficiency and overall physiological function. Consequently, their use in equine diets has become increasingly popular (31, 32). Corn, soybean, and canola oils are among the most frequently incorporated lipid sources, either as feed supplements or as top-dress additives for grains (31, 33). Despite the growing interest in alternative lipid sources, no studies to date have investigated the effects of hemp supplementation on the reproductive performance of donkey stallions.

In recent decades, donkey farming has attracted growing interest from both researchers and consumers, largely due to the recognized health benefits of donkey milk (34, 35). The rediscovery of its nutritional and therapeutic properties has played a key role in preserving preservation and, in some cases, restoring several native donkey breeds (36, 37). Additionally, policies promoting farm diversification and supporting the conservation of local animal populations have further encouraged the expansion of donkey farming. These animals are particularly well-suited to marginal areas that would otherwise be unsuitable for other types of livestock production (38). The increasing demand for donkey-derived products has led many farmers to expand their herds and adopt modern technologies, including milking systems (35). In this context, developing strategies to enhance the reproductive performance of donkeys is crucial to ensuring sustainable herd growth and the long-term viability of these animals. Currently, one of the most commonly used breeds for milk production is the Martina Franca donkey (38). Native to the rural areas of the Apulia region in Italy, this breed is characterized by its tall stature, exceptional robustness, and docile temperament (39). These traits have contributed to its expansion beyond its region of origin, with increasing presence in central and northern Italy (40).

This study represents the first investigation into the effects of hemp-based polyunsaturated fatty acid supplementation on the membrane lipid profiles and reproductive performance of Martina Franca jacks.

## 2 Materials and methods

The study was conducted at the experimental stables of the University of Teramo, located in Bellante (Teramo, Italy), over a period of 90 days. Ethical approval for the experimental procedures was obtained from the Ethical Animal Care and Use Committee of the University of Teramo (Protocol No. 18532 of 28/06/2022), and all activities were carried out in accordance with Directive 2010/63/EU.

### 2.1 Animals and diets

Twelve Martina Franca donkey jack (average age: 7 ± 3 years; mean body weight: 333 ± 51 kg) were enrolled in this study. Prior to the study, the donkeys underwent a comprehensive clinical examination to confirm they were in good health status. Additionally, inclusion criteria required a favorable body condition score (BCS; average 2.8 ± 0.4), assessed by visual appraisal and palpation was obtained independently by two technicians, but final classification was decided by consensus if there was any discrepancy, and the maintenance of optimal health throughout the trial period. To ensure this, the animals were monitored weekly by trained personnel who assessed various health indicators. These assessments included physical condition, skin cleanliness, eye health (evaluating for sunken or dull eyes, and the tendency to keep eyes closed or partially closed), salivation, ear positioning during inspection, and respiratory patterns (such as tachypnea, hyperpnea, or dyspnea). Additionally, feed and water intake, social interactions (e.g., signs of fleeing or hiding), the presence of stereotypies or aggression, posture, lameness or uncoordinated movements, and the presence of skin or ocular lesions were all evaluated. The presence of blood in the feces, fecal consistency, and cleanliness of the stall were also assessed. Each of these criteria was scored on a scale from 1 to 6, with 1 indicating abnormal or pathological conditions and 6 indicating normal conditions.

All jacks were housed in individual paddocks (each at least 160 m^2^) with appropriate shelter, water troughs, and feeders. For the experimental trial, the animals were divided into two groups: the treatment group (TRT) and the control group (CTR). Each donkey had free access to an *ad libitum* amount (41) of clean drinking water throughout the study. The donkeys were fed according to the nutritional requirements outlined in the Nutrient Requirements of Horses (42), taking into account their weight, age, and activity level. The trial lasted for 3 months, during which the stallions received an isoproteic diet. The treatment group’s diet was enriched with hemp oil, while the control group did not receive this supplement. All animals had *ad libitum* access to mixed hay (2% of body weight), and complete feed was provided twice daily. The ingredients of the complete feed are listed in Table 1. To minimize the risk of colic and ensure the animals’ welfare, dietary changes were introduced gradually over a 15-day period, allowing a smooth transition to the new feeding regime (43, 44). During this period, animals were closely monitored for any signs of digestive discomfort or behavioral changes, and no adverse effects were observed. This approach was aligned with ethical guidelines for animal welfare in nutritional trials.

**Table 1:** Ingredients (kg/head/day) and chemical composition of the experimental feeds.

	CTR	TRT
Oat meal	0.64	0.64
Faba bean meal	0.20	0.20
Soybean hulls	0.66	0.66
Hemp oil	0.00	0.08
Min-vit supplement	0.06	0.06

### 2.2 Sampling operations

#### 2.2.1 Feed sampling and chemical analysis

During the experimental period, hays and feed samples were collected once a week for qualitative and chemical-nutritional evaluation. Additionally, the quality of the hay was assessed following the guidelines of Cavallini et al. (23) to ensure the absence of molds and spores. All collected samples were immediately transported to the laboratory for dry matter (DM) and chemical analysis, using detailed methodologies for feed analysis previously published by other authors (45, 46).

#### 2.2.2 Semen sampling and analysis

Each stallion selected for this research project had been approved for assisted reproduction and had previously participated in breeding programs. As a result, the donkeys underwent a training protocol for semen collection procedures. Semen was collected weekly using a Missouri artificial vagina, with each stallion assigned a specific device at the start of the breeding season. During collection, an in-line gel filter (Minitüb, Tiefenbach, Germany) was used, and a jenny in natural or induced estrus was present to stimulate the stallion’s copulatory response. After collection, semen samples were promptly sent at room temperature to the laboratory, located in the same facility, for semen analysis. The total semen volume (vol. tot) was measured using a graduated cylinder immediately after collection, while the gel-free volume (vol. gf) was determined by filtering the sample through sterile gauze into a graduated test tube. Sperm concentration (conc.) and the proportion of nonviable spermatozoa (death) were assessed using an automated sperm counting system [Nucleo-Counter SP 100TM, ChemoMetec, Allerod, Denmark; (47)]. Motility parameters (total and progressive motility) and sperm morphology (morph.) were evaluated with a computer-assisted semen analysis system (CASA IVOS II, Hamilton Thorne, Beverly, MA, United States), following the standardized settings provided by the manufacturer, as described by Contri et al. (48). To analyze the semen lipidomic profile, sperm cells were separated from the seminal plasma through two consecutive centrifugations (2,500 rpm for 10 min). After removing the seminal plasma, the cells were washed with PBS (0.5 mL, pH 7.8) twice (2,500 rpm × 5 min each). The sperm cells were then resuspended in pure water (18 mQ) to achieve a concentration of 6 × 10^6^ cells per mL. This suspension was used to extract membrane lipids, employing a 2:1 chloroform mixture as the organic phase, following the Folch method (49). The organic layer was subsequently extracted, and the sample was dried under vacuum.

#### 2.2.3 Blood sampling and analysis

Blood samples (1 mL each) were collected every 15 days. The samples were drawn from the jugular vein by a trained technician and collected in tubes containing ethylenediaminetetraacetic acid (EDTA) as a tripotassium salt. To analyze the erythrocyte lipidomic profile, erythrocytes were isolated from whole blood collected in EDTA tubes. The procedure for membrane lipidome analysis was conducted as previously outlined by Prasinou et al. (50). In brief, starting with a 1 mL whole blood sample, erythrocytes were separated from plasma by two consecutive centrifugations (3,000 g × 5 min, each), followed by a washing step with phosphate buffer (0.5 mL) two times (3,000 g × 5 min, each) and finally obtaining the erythrocyte membrane pellet by centrifugation (15,000 g × 15 min). The resulting pellet was then resuspended in pure water and used for phospholipid extraction using a 2:1 chloroform mixture as the organic phase, following the same methodology as described for the semen lipidomic analysis.

#### 2.2.4 Gas chromatography

To assess the effectiveness of the lipid extraction, thin layer chromatography (TLC) was carried out using a chloroform/methanol/water solvent system (65:25:4) as described by Fuchs et al. (51). The extracted phospholipids were then transesterified at room temperature for 10 min using 0.5 M KOH in methanol to produce the corresponding fatty acid methyl esters (FAMEs). This chemical conversion was performed according to established protocols, with careful monitoring to prevent oxidative or degradative reactions that could alter the fatty acid profile. Each sample was analyzed in duplicate, with the entire procedure repeated twice to ensure the accuracy and reliability of the results.

Initially, gas chromatography (GC) analysis was conducted on commercially available reference standards for each of the 9 selected fatty acids. Calibration curves for quantitative analysis were generated for each chromatogram peak, as illustrated in Prasinou et al. (52). The FAME mixture obtained from both sperm and erythrocyte membrane pellets was dissolved in 20 μL of n-hexane, and 1 μL of this solution was directly injected into an Agilent 7890B GC system equipped with a flame ionization detector and a DB-23 (50%-Cyanopropyl)-methylpolysiloxane capillary column (60 m, 0.25 mm i.d., 0.25 μm film thickness). The GC temperature program started at 165°C, maintained for 3 min, followed by a gradual increase of 1°C/min until reaching 195°C, which was held for 40 min. The temperature was then increased by 10°C/min up to 240°C and held for 10 min. Hydrogen was used as the carrier gas, with a constant pressure of 16.482 psi. FAMEs were identified by comparing their retention times with those of standard references, which were either commercially sourced or synthesized.

### 2.3 Statistical analysis

The effects of hemp-based polyunsaturated fatty acid supplementation on the semen parameters, membrane lipid profiles, and reproductive performance in Martina Franca jacks were evaluated across different time points: Day 15 (D15), Day 45 (D45), and Day 60 (D60). The normal distribution of the data was tested by the Shapiro–Wilk normality test according to Ferlizza et al. (53). Measurements were not normally distributed, and they were therefore normalized by Box–Cox transformation as reported in Dini et al. (54). Repeated-measures linear mixed-effects models were constructed as previously (55) according to the sampling time points performed. Model fixed effect was the group while each subject was the experimental unit. After the analysis, normal distribution of the data was checked again for the resulting residuals. A bivariate matrix with Pearson correlation was calculated to evaluate the relationship between ration and or semen and or blood parameters, according to Bordin et al. (56).

## 3 Results

Regarding semen parameters (Table 2), the gel-free volume of semen showed a significant decrease following supplementation. The control group (CTR) exhibited a gel-free volume of 64.17 mL, which significantly reduced to 45.18 mL at D15 and remained stable at 45.90 mL by D45. A further reduction was observed at D60, with the volume reaching 28.20 mL (*p* < 0.0001). Sperm concentration also demonstrated a significant decline, starting from 632.89 million/mL in the control group and decreasing to 371.69 million/mL at D15 and 343.10 million/mL at D45. By D60, the concentration showed a slight recovery to 407.28 million/mL; however, it remained significantly lower than the control (p < 0.0001). These changes suggest a notable impact of the hemp oil supplementation on semen volume and concentration. Sperm motility displayed a different trend, with a slight increase from the control value of 92.61 to 95.08% at D15, and a further improvement to 96.63% by D60 (*p* = 0.05). Progressive motility, however, did not show significant changes across the supplementation periods. Normal morphology of the sperm significantly improved from 96.58% in the control group to 98.12% at D15, remained consistent at 96.26% at D45, and further increased to 98.85% by D60 (*p* = 0.04).

**Table 2:** Semen parameters of Martina Franca jacks after different timepoints^a^ of inclusion of hemp oil.

Item	CTR	D15	D45	D60	SEM	*p*-value
Total volume (mL)	79.17	72.97	68.98	64.77	5.91	0.19
Gel-free volume (mL)	64.17^A^	45.18^B^	45.90^B^	28.20^C^	5.50	<0.0001
Concentration (mln/mL)	632.89^A^	371.69^B^	343.10^B^	407.28^B^	50.74	<0.0001
Dead (%)	12.61	13.16	11.08	14.06	2.11	0.56
Motility (%)	92.61^B^	95.08^AB^	93.06^B^	96.63^A^	1.18	0.05
Progressive Motility (%)	65.89^A^	66.66^AB^	67.38^B^	63.64^A^	2.49	0.62
Normal morphology (%)	96.58^B^	98.12^AB^	96.26^B^	98.85^A^	0.77	0.04

a D15: results after 15 days of inclusion; D45: results after 45 days of inclusion; D60: results after 15 days of inclusion.

^A,B,C^ Least squares means with different superscript letters within a row are significantly different (*p* < 0.05).

The membrane lipid profiles of spermatozoa (Table 3) revealed significant changes in fatty acid composition. The proportion of C18:0 decreased significantly from 10.06% in the control group to 7.88% at D45, with a partial recovery to 9.14% at D60 (*p* = 0.001). In contrast, the proportion of C18:2 9c 12c significantly increased from 4.54% in the control group to 5.63% at D45 and 5.97% at D60 (*p* = 0.003). Moreover, the EPA content showed a significant increase after supplementation, rising from 0.08% in the control group to 0.16% at D45 and 0.20% at D60 (*p* < 0.0001). Significant alterations were also observed in the overall lipid profile indices. The ω3/(ω3 + ω6) ratio and the unsaturation index both decreased significantly after 45 days of supplementation, with the unsaturation index dropping from 233.53 in the control group to 87.65 at D45 (*p* < 0.0001). The peroxidation index also showed a significant reduction, suggesting decreased susceptibility to lipid peroxidation (*p* < 0.0001).

**Table 3:** Fatty acids (% of the Found μg/mL) of spermatozoa membrane glycerophospholipids after different timepoints^a^ of inclusion of hemp oil.

FAME (%μg/mL)	D0	D15	D45	D60	SEM	*p*-value
C14:0	4.51	4.16	4.81	4.54	0.27	0.45
C16:0	30.45	30.19	30.76	29.15	0.83	0.50
C16:1 9c	0.13	0.10	0.10	0.10	0.03	0.81
C18:0	10.06^A^	11.08^A^	7.88^B^	9.14^AB^	0.51	0.001
C18:1 9c	2.73	2.80	2.79	3.21	0.18	0.19
C18:1 11c	3.32^AB^	2.81^B^	3.68^AB^	3.95^A^	0.25	0.03
C18:2 9c, 12c	4.54^B^	4.37^B^	5.63^AB^	5.97^A^	0.32	0.003
C20:3 8c, 11c, 14c	2.13	2.28	2.15	2.06	0.18	0.88
C20:4 5c, 8c, 11c, 14c	6.54	5.81	5.10	5.38	0.53	0.19
EPA	0.08^B^	0.07^B^	0.16^A^	0.20^A^	0.01	<0.0001
DPA n-6	27.63^A^	27.77^A^	29.67^A^	29.36^A^	0.70	0.04
DHA	7.78^AB^	8.59^A^	7.16^AB^	6.80^B^	0.43	0.04
SFA	45.10	45.55	43.53	42.89	0.80	0.07
MUFA	6.20^AB^	5.70^B^	6.56^AB^	7.27^A^	0.32	0.02
ω3	7.85	8.66	7.33	6.99	0.43	0.07
ω6	40.96^BC^	40.24^C^	42.57^AB^	42.86^A^	0.71	0.04
PUFA	48.80^A^	48.82^A^	20.10^B^	20.36^B^	0.85	<0.0001
SFA/MUFA	7.57^A^	8.09^A^	6.79^AB^	5.99^B^	0.43	0.01
ω6/ω3	5.44	4.86	5.90	6.26	0.35	0.07
ω3/(ω3 + ω6)	16.00^B^	17.66^B^	36.83^A^	34.52^A^	1.33	<0.0001
Unsaturation Index	233.53^A^	234.90^A^	87.65^B^	88.18^B^	3.52	<0.0001
Peroxidation Index	264.11^A^	267.66^A^	87.51^B^	86.53^B^	4.07	<0.0001

a D15: results after 15 days of inclusion; D45: results after 45 days of inclusion; D60: results after 15 days of inclusion.

^A,B,C^ Least squares means with different superscript letters within a row are significantly different (*p* < 0.05).

In erythrocyte membrane lipid profiles (Table 4), the study found that the level of C16:0 significantly decreased from 10.79% in the control group to 9.27% at D45 (*p* = 0.05). Similarly, the proportion of C18:1 9c increased significantly from 29.39% in the control group to 32.28% at D60 (*p* = 0.001). The ω6/ω3 ratio showed a significant increase from 211.63 in the control group to 259.22 at D60 (*p* = 0.03). The Pearson correlation coefficients among semen parameters are presented in Table 5. Gel-free volume was strongly positively correlated with total volume (*r* = 0.82), indicating a substantial relationship between these variables. Weak to negligible correlations were observed between concentration and other parameters, including total volume (*r* = 0.05) and gel-free volume (*r* = 0.23). Motility showed a weak negative correlation with gel-free volume (*r* = −0.06) and concentration (*r* = −0.11), while progressive motility displayed a modest positive correlation with gel-free volume (*r* = 0.37). Dead percentage exhibited weak negative correlations with total volume (*r* = −0.22) and gel-free volume (*r* = −0.30). A strong positive correlation was noted between motility and normal morphology (*r* = 0.80).

**Table 4:** Fatty acids (% of the Found μg/mL) of erythrocyte membrane glycerophospholipids after different timepoints^a^ of inclusion of hemp oil.

FAME (%μg/mL)	D0	D15	D45	D60	SEM	*p*-value
C16:0	10.79^A^	9.60^AB^	9.27^B^	9.36^B^	0.47	0.05
C16:1 9c	0.89	0.83	0.91	1.10	0.10	0.31
C18:0	9.28	8.22	9.07	8.53	0.42	0.12
C18:1 9c	29.39^B^	28.73^B^	28.39^B^	32.28^A^	0.61	0.001
C18:1 11c	0.89	0.85	1.01	0.99	0.06	0.09
C18:2 9c, 12c	46.35^B^	49.57^A^	49.45^A^	45.77^B^	0.72	0.001
C20:3 8c, 11c, 14c	0.16^B^	0.17^AB^	0.20^A^	0.19^AB^	0.01	0.03
C20:4 5c, 8c, 11c, 14c	1.51	1.79	1.41	1.37	0.12	0.09
EPA	0.18^AB^	0.21^A^	0.18^AB^	0.16^B^	0.01	0.01
DHA	0.05^A^	0.04^AB^	0.04^AB^	0.03^B^	0.01	0.05
SFA	19.99^A^	17.74^B^	18.34^AB^	17.87^B^	0.70	0.02
MUFA	31.35^B^	30.43^B^	30.32^B^	34.47^A^	0.73	0.01
ω3	0.23^A^	0.25^A^	0.22^AB^	0.20^B^	0.01	0.01
ω6	48.01^B^	51.54^A^	51.05^A^	47.32^B^	0.79	0.001
PUFA	48.25^B^	51.80^A^	51.26^A^	47.52^B^	0.80	0.001
SFA/MUFA	0.64^A^	0.59^AB^	0.60^AB^	0.52^B^	0.03	0.03
ω6/ω3	211.63^B^	208.37^AB^	239.06^AB^	259.22^A^	14.00	0.03
ω3/(ω3 + ω6)	0.48^A^	0.48^AB^	0.42^AB^	0.41^B^	0.02	0.02
Unsaturation Index	132.26^B^	138.66^A^	136.66^AB^	133.36^B^	1.50	0.004
Peroxidation Index	54.97^B^	59.46^A^	57.60^AB^	53.72^B^	1.09	0.003

a D15: results after 15 days of inclusion; D45: results after 45 days of inclusion; D60: results after 15 days of inclusion.

^A,B,C^ Least squares means with different superscript letters within a row are significantly different (*p* < 0.05).

**Table 5:** Pearson correlations between semen parameters.

	Total volume	Gel-free volume	Concentration	Motility	Progressive Motility	Dead %	Normal morphology
Total volume							
Gel-free volume	0.82*						
Concentration	0.05	0.23					
Motility	−0.03	−0.06	−0.11				
Progressive Motility	0.32*	0.37*	−0.19	0.10			
Dead %	−0.22	−0.30*	−0.12	−0.12	−0.07		
Normal morphology	−0.16	−0.18	0.05	0.80*	0.04	0.02	

*<0.05.

Significant correlations were observed between dietary fatty acid profiles and spermatozoa membrane glycerophospholipids (Table 6). Among the most relevant associations, PUFA balance ω3/(ω3 + ω6) (*r* = 0.53, *p* = 0.01) showed the highest positive correlations, while the ω6/ω3 ratio was negatively correlated (*r* = −0.49, *p* = 0.01). For erythrocyte membrane glycerophospholipids (Table 7), stearic acid (C18:0, *r* = 0.50, p = 0.01), and PUFA (*r* = 0.98, *p* = 0.01) exhibited strong positive correlations with dietary fatty acids, whereas EPA (*r* = −0.78, *p* = 0.01) and the PUFA balance ω3/(ω3 + ω6) (*r* = −0.91, *p* = 0.01) showed negative correlations. The relationships between semen parameters and spermatozoa membrane fatty acids (Table 8) revealed that PUFA, along with the unsaturation index (UI) and peroxidation index (PI), were correlated with sperm concentration (ranging from *r* = 0.48 to *r* = 0.69, *p* < 0.05). For erythrocyte membrane fatty acids (Table 9), stearic acid (C18:0) was positively correlated with gel-free volume (*r* = 0.63, *p* < 0.05). PUFA, UI, and PI exhibited strong associations with sperm concentration (ranging from *r* = 0.44 to *r* = 0.69, *p* < 0.05). Finally, comparative analyses of fatty acid profiles between spermatozoa and erythrocyte membrane glycerophospholipids (Tables 10–12) showed that ω3 levels in spermatozoa were positively correlated with PUFA levels in erythrocytes (*r* = 0.53, *p* < 0.05).

**Table 6:** Pearson correlations between fatty acid profile in diet and fatty acids of spermatozoa membrane glycerophospholipids.

	Ration	*p*-value
16:00	0,3,713	0,03
16:1 9c	−0,2,524	0,16
18:00	0,0538	0,77
18:1 9c	−0,2,638	0,14
18:1 11c	−0,3,799	0,03
18:2 9c, 12c	0,0072	0,97
20:3 8c, 11c, 14c	−0,3,472	0,05
20:4 5c, 8c, 11c, 14c	0,3,324	0,06
EPA	0,4,044	0,02
DHA	0,3,932	0,02
SFA	0,2,745	0,12
MUFA	−0,3,024	0,09
ω3	0,4,852	0,01
ω6	0,0442	0,81
PUFA	0,0505	0,78
SFA/MUFA	0,3,388	0,05
ω6/ω3	−0,4,918	0,01
PUFA balance ω3/(ω3 + ω6)	0,5,256	0,01
UI	−0,0413	0,82
PI	0,1,556	0,39

**Table 7:** Pearson correlations between fatty acid profile in diet and fatty acids of erythrocyte membrane glycerophospholipids.

	Ration	*p*-value
14:00	−0,1796	0,25
16:00	0,0799	0,61
16:1 9c	0,1,011	0,52
18:00	0,503	0,01
18:1 9c	−0,2,104	0,18
18:1 11c	−0,3,995	0,01
18:2 9c, 12c	−0,5,531	0,01
20:3 8c, 11c, 14c	0,0766	0,63
20:4 5c, 8c, 11c, 14c	0,2,995	0,05
EPA	−0,7,761	0,01
DPA n-6	−0,4,014	0,01
DHA	0,378	0,01
SFA	0,393	0,01
MUFA	−0,4,057	0,01
ω3	0,3,497	0,02
ω6	−0,4,266	0,01
PUFA	0,9,849	0,01
SFA/MUFA	0,4,571	0,01
ω6/ω3	−0,3,682	0,01
PUFA balance ω3/(ω3 + ω6)	−0,9,104	0,01
UI	0,9,903	0,01
PI	0,9,913	0,01

**Table 8:** Pearson correlation between semen parameters and fatty acids (% of the Found μg/mL) of spermatozoa membrane glycerophospholipids.

	Total volume	Gel-free volume	Concentration	Motility	Progressive Motility	Dead %	Normal morphology
C14:0	−0.18	−0.13	−0.08	−0.04	−0.01	0.02	0.07
C16:0	0.16	0.15	0.03	−0.30*	0.04	0.08	−0.27
C16:1 9c	0.02	0.02	−0.04	0.05	−0.05	−0.16	0.09
C18:0	0.39*	0.47*	0.24	0.43*	0.33*	−0.20	0.32*
C18:1 9c	0.01	−0.06	−0.03	0.27	−0.13	−0.10	0.23
C18:1 11c	−0.24	−0.22	−0.06	0.29*	−0.16	0.00	0.22
C18:2 9c, 12c	−0.37*	−0.49*	−0.20	−0.04	−0.24	0.26	−0.18
C20:3 8c, 11c, 14c	0.00	0.01	−0.07	−0.25	−0.18	0.10	−0.17
C20:4 5c, 8c, 11c, 14c	0.04	0.11	0.05	−0.26	−0.06	−0.11	−0.20
EPA	−0.17	−0.37*	−0.32*	0.27	0.02	0.11	0.17
DPA n-6	−0.18	−0.24	−0.09	0.19	0.03	0.00	0.32*
DHA	0.01	0.05	0.05	−0.16	−0.06	−0.02	−0.27
SFA	0.38*	0.44*	0.17	−0.02	0.27	−0.05	−0.04
MUFA	−0.19	−0.22	−0.07	0.39*	−0.21	−0.07	0.32*
ω3	0.00	0.03	0.03	−0.15	−0.06	−0.02	−0.26
ω6	−0.35*	−0.41*	−0.18	−0.08	−0.18	0.09	0.04
PUFA	0.46*	0.68*	0.48*	−0.28	0.22	−0.17	−0.24
SFA/MUFA	0.25	0.33*	0.11	−0.28	0.23	0.02	−0.20
ω6/ω3	−0.13	−0.17	0.01	0.08	−0.07	0.06	0.25
Unsaturation Index	0.47*	0.69*	0.48*	−0.27	0.22	−0.19	−0.22
Peroxidation Index	0.47*	0.69*	0.48*	−0.26	0.23	−0.19	−0.22

*<0.05.

**Table 9:** Pearson correlation between semen parameters and fatty acids (% of the Found μg/mL) of erythrocyte membrane glycerophospholipids.

	Total volume	Gel-free volume	Concentration	Motility	Progressive motility	Dead %	Normal morphology
C16:0	−0.02	0.29	0.49*	−0.11	0.04	−0.14	−0.05
C16:1 9c	−0.15	−0.09	0.11	0.20	0.03	0.12	0.12
C18:0	0.46*	0.63*	0.14	−0.07	0.14	−0.21	−0.15
C18:1 9c	0.13	0.10	−0.19	0.00	0.21	0.28	−0.18
C18:1 11c	0.12	0.05	−0.09	0.15	0.08	0.07	−0.05
C18:2 9c, 12c	−0.28	−0.44*	−0.10	0.04	−0.21	−0.06	0.18
C20:3 8c, 11c, 14c	−0.35*	−0.57*	−0.30	0.08	−0.26	0.17	0.23
C20:4 5c, 8c, 11c, 14c	0.05	−0.05	−0.12	0.06	−0.17	−0.02	0.19
EPA	−0.22	−0.20	0.01	−0.17	−0.13	−0.19	−0.02
DHA	−0.11	−0.16	0.00	−0.38*	−0.23	0.08	−0.13
SFA	0.28	0.57*	0.37*	−0.10	0.12	−0.22	−0.13
MUFA	0.10	0.08	−0.16	0.04	0.20	0.28	−0.15
ω3	−0.21	−0.22	0.01	−0.29	−0.20	−0.11	−0.07
ω6	−0.25	−0.42*	−0.11	0.05	−0.21	−0.06	0.19
PUFA	−0.26	−0.42*	−0.11	0.04	−0.22	−0.06	0.19
SFA/MUFA	0.17	0.43*	0.44*	−0.10	−0.01	−0.34*	0.01
ω6/ω3	0.08	−0.05	−0.10	0.34*	0.08	0.22	0.23
ω3/(ω3 + ω6)	−0.09	0.00	0.08	−0.37*	−0.11	−0.10	−0.20
Unsaturation Index	−0.26	−0.48*	−0.22	0.07	−0.20	0.05	0.19
Peroxidation Index	−0.20	−0.36*	−0.13	0.04	−0.22	−0.05	0.20

*<0.05.

**Table 10:** Pearson correlation between fatty acids (% of the Found μg/mL) of spermatozoa membrane glycerophospholipids.

	C14	C16	C16:1 9c	C18	C18:1 9c	C18:1 11c	C18:2 9c, 12c	C20:3 8c, 11c, 14c	C20:4 5c, 8c, 11c, 14c	EPA	DPA n-6	DHA	SFA	MUFA	ω3	ω6	PUFA	UI	PI
C14	0.07																		
C16	−0.27	−0.33*																	
C16:1 9c	0.09	0.03	−0.08																
C18	0.32*	−0.18	−0.24	0.07															
C18:1 9c	0.23	−0.22	−0.22	0.09	0.23														
C18:1 11c	0.22	0.15	−0.50*	0.00	−0.08	0.00													
C18:2 9c, 12c	−0.18	−0.01	−0.16	0.07	−0.41*	−0.13	0.27												
C20:3 8c, 11c, 14c	−0.17	−0.01	0.18	−0.14	−0.37*	−0.15	−0.15	0.12											
C20:4 5c, 8c, 11c, 14c	−0.20	−0.24	−0.10	0.20	0.04	−0.09	−0.01	0.05	0.06										
EPA	0.17	0.10	−0.05	−0.02	−0.14	0.02	0.13	0.32*	−0.18	−0.19									
DPA n-6	0.32*	0.34*	−0.48*	−0.13	−0.10	0.11	0.17	−0.16	−0.17	−0.49*	0.17								
DHA	−0.27	0.11	−0.07	−0.11	−0.16	−0.13	−0.09	−0.01	−0.03	−0.14	−0.19	−0.16							
SFA	−0.04	−0.18	0.77*	−0.02	0.38*	−0.12	−0.53*	−0.44*	−0.07	−0.15	−0.12	−0.47*	−0.15						
MUFA	0.32*	0.01	−0.54*	0.14	0.07	0.57*	0.82*	0.15	−0.22	−0.04	0.11	0.19	−0.15	−0.50*					
ω3	−0.26	0.11	−0.08	−0.11	−0.17	−0.13	−0.08	0.01	−0.04	−0.15	−0.13	−0.15	1.00*	−0.16	−0.15				
ω6	0.04	0.15	−0.59*	0.01	−0.38*	−0.06	0.26	0.42*	0.20	0.27	0.15	0.53*	−0.27	−0.82*	0.18	−0.26			
PUFA	−0.24	−0.12	0.00	0.10	0.45*	−0.18	−0.18	−0.41*	−0.03	0.43*	−0.65*	−0.39*	0.22	0.28	−0.24	0.19	−0.31*		
UI	−0.22	−0.11	0.00	0.10	0.47*	−0.16	−0.18	−0.45*	−0.06	0.39*	−0.65*	−0.36*	0.24	0.29*	−0.23	0.20	−0.33*	−0.26	
PI	−0.22	−0.11	0.01	0.09	0.48*	−0.16	−0.19	−0.47*	−0.07	0.37*	−0.65*	−0.35*	0.25	0.31*	−0.24	0.21	−0.35*	−0.27	

*<0.05.

**Table 11:** Pearson correlation between fatty acids of erythrocyte membrane glycerophospholipids.

	C16:0	C16:1 9c	C18:0	C18:1 9c	C18:1 11c	C18:2 9c,12c	C20:3 8c, 11c, 14c	C20:4 5c, 8c, 11c, 14c	EPA	DHA	SFA	MUFA	ω3	ω6	PUFA	UI
C16:0																
C16:1 9c	0.46*															
C18:0	0.35*	0.03														
C18:1 9c	−0.04	0.29	0.19													
C18:1 11c	0.08	0.19	0.12	0.38*												
C18:2 9c, 12c	−0.51*	−0.46*	−0.67*	−0.75*	−0.35*											
C20:3 8c, 11c, 14c	−0.49*	−0.20	−0.67*	−0.42*	−0.24	0.70*										
C20:4 5c, 8c, 11c, 14c	−0.36*	−0.31	−0.35*	−0.49*	−0.45*	0.55*	0.74*									
EPA	−0.04	−0.09	−0.33*	−0.42*	−0.41*	0.40*	0.57*	0.52*								
DHA	−0.17	−0.31	−0.29	−0.36*	−0.19	0.42*	0.46*	0.43*	0.33*							
SFA	0.80*	0.28	0.85*	0.10	0.12	−0.72*	−0.71*	−0.43*	−0.24	−0.28						
MUFA	0.03	0.41*	0.19	0.99*	0.44*	−0.78*	−0.43*	−0.52*	−0.43*	−0.39*	0.14					
ω3	−0.11	−0.20	−0.38*	−0.48*	−0.40*	0.49*	0.64*	0.59*	0.91*	0.69*	−0.31	−0.50*				
ω6	−0.52*	−0.46*	−0.67*	−0.75*	−0.38*	1.00*	0.74*	0.63*	0.43*	0.44*	−0.73*	−0.78*	0.52*			
PUFA	−0.51*	−0.46*	−0.67*	−0.75*	−0.38*	1.00*	0.75*	0.63*	0.44*	0.44*	−0.72*	−0.78*	0.53*	1.00*		
UI	−0.65*	−0.42*	−0.78*	−0.56*	−0.33*	0.95*	0.82*	0.68*	0.43*	0.44*	−0.87*	−0.59*	0.52*	0.96*	0.96*	
PI	−0.51*	−0.45*	−0.64*	−0.74*	−0.43*	0.95*	0.81*	0.78*	0.51*	0.49*	−0.71*	−0.77*	0.61*	0.98*	0.98*	0.96*

*<0.05.

**Table 12:** Pearson correlation between fatty acids (% of the Found μg/mL) of spermatozoa membrane glycerophospholipids and fatty acids of erythrocyte membrane glycerophospholipids.

	C14	C16	C16:1 9c	C18	C18:1 9c	C18:1 11c	C18:2 9c, 12c	C20:3 8c, 11c, 14c	C20:4 5c, 8c, 11c, 14c	EPA	DPA n-6	DHA	SFA	MUFA	ω3	ω6	PUFA	UI	PI
C16:0	−0.10	−0.03	−0.02	0.41*	−0.05	−0.25	−0.24	0.08	0.16	−0.45*	−0.22	0.15	0.26	−0.24	0.13	−0.23	0.47*	0.47*	0.48*
C16:1 9c	−0.28	0.04	−0.11	0.13	0.21	−0.25	0.13	0.18	−0.10	−0.04	−0.18	0.21	0.05	−0.09	0.21	−0.15	−0.04	−0.05	−0.04
C18:0	−0.13	−0.06	0.21	0.32*	0.24	−0.16	−0.27	−0.09	0.31	−0.30	−0.19	0.00	0.14	0.02	−0.01	−0.15	0.42*	0.43*	0.42*
C18:1 9c	−0.01	−0.11	0.11	0.03	0.17	0.12	0.23	−0.02	−0.04	−0.02	−0.15	0.12	−0.11	0.21	0.12	−0.08	0.14	0.13	0.13
C18:1 11c	−0.17	0.19	−0.05	0.09	−0.04	0.14	0.16	−0.28	−0.23	0.04	−0.07	−0.09	0.22	0.09	−0.09	−0.23	−0.16	−0.16	−0.15
C18:2 9c, 12c	0.14	0.10	−0.14	−0.32*	−0.24	0.10	0.07	0.00	−0.13	0.33*	0.27	−0.16	−0.09	−0.07	−0.15	0.23	−0.44*	−0.44*	−0.44*
C20:3 8c, 11c, 14c	0.11	0.08	−0.19	−0.38*	0.02	0.22	0.08	0.14	−0.29	0.32*	0.22	−0.05	−0.17	0.18	−0.03	0.11	−0.59*	−0.58*	−0.58*
C20:4 5c, 8c, 11c, 14c	0.07	−0.09	−0.04	−0.05	0.09	0.12	−0.18	0.17	−0.11	0.03	0.17	0.01	−0.12	0.15	0.01	0.06	−0.10	−0.09	−0.09
EPA	−0.13	0.01	−0.07	−0.17	−0.05	0.00	−0.25	0.16	0.15	−0.14	0.07	0.18	−0.17	−0.04	0.17	0.09	0.01	0.02	0.03
DHA	0.03	0.32*	−0.05	−0.17	−0.05	−0.24	−0.23	0.09	0.22	−0.05	−0.20	0.08	0.23	−0.23	0.07	−0.16	0.02	0.03	0.03
SFA	−0.14	−0.05	0.12	0.44*	0.13	−0.25	−0.31	−0.01	0.29	−0.45*	−0.25	0.09	0.24	−0.12	0.06	−0.22	0.54*	0.54*	0.55*
MUFA	−0.06	−0.09	0.08	0.05	0.18	0.08	0.24	−0.01	−0.07	−0.02	−0.17	0.14	−0.08	0.18	0.14	−0.10	0.11	0.11	0.10
ω3	−0.08	0.14	−0.07	−0.21	−0.06	−0.10	−0.29	0.16	0.21	−0.13	−0.03	0.17	−0.03	−0.13	0.16	0.00	0.02	0.03	0.03
ω6	0.13	0.08	−0.14	−0.31	−0.21	0.11	0.05	0.02	−0.14	0.31	0.27	−0.15	−0.10	−0.05	−0.14	0.22	−0.43*	−0.43*	−0.42*
PUFA	0.13	0.09	−0.14	−0.31	−0.21	0.11	0.04	0.02	−0.14	0.31	0.27	−0.15	−0.10	−0.05	−0.13	0.22	−0.42*	−0.42*	−0.42*
UI	0.14	0.06	−0.14	−0.36	−0.17	0.18	0.12	0.04	−0.20	0.36*	0.28	−0.12	−0.17	0.04	−0.10	0.23	−0.48*	−0.48*	−0.47*
PI	0.12	0.05	−0.12	−0.27	−0.14	0.12	−0.02	0.07	−0.13	0.25	0.26	−0.11	−0.11	0.00	−0.10	0.19	−0.37*	−0.36*	−0.36*

*<0.05.

## 4 Discussions

This study represents the first investigation into the effects of hemp-based PUFA supplementation on membrane lipid profiles and reproductive performance in Martina Franca jacks. The findings provide novel insights into the interaction between diet and reproductive function, supporting the hypothesis that dietary modulation of fatty acid profiles can influence reproductive health. While the role of nutrition in regulating female reproductive function is well-documented and widely recognized [e.g., the effects of sudden changes in body condition in dairy cows; (57)], its significance for male fertility is equally substantial, as highlighted by several studies [e.g., (58–60)].

The effect of diet on sperm quality is shaped by both quantitative and qualitative aspects, including the macronutrient composition (primarily fats) and the specific proportions of carbohydrates, proteins, and fatty acids (61). Among these, fatty acids play a crucial role in sperm physiology by regulating membrane fluidity, acrosome reaction, motility, and viability (62). Sperm membrane lipids are fundamental for preserving structural integrity and facilitating membrane fusion events during fertilization (11, 62, 63). Notably, PUFAs are especially important, as they integrate into the sperm plasma membrane, enhancing its flexibility, preserving functional stability, and increasing the osmotic resistance of the acrosome membrane. Additionally, they provide a protective effect against physiological stressors and thermal fluctuations during cryopreservation (62, 64). Several studies have demonstrated that optimal PUFA levels in semen extenders can enhance sperm antioxidant capacity, improve DNA integrity (65), and mitigate oxidative stress (66). These findings underscore the potential of dietary interventions, particularly through PUFA supplementation, to support sperm function and overall reproductive efficiency. However, excessive intake of high-calorie diets, particularly those rich in SFAs and trans fats, can have a detrimental effect on sperm quality, primarily by increasing oxidative stress—a key factor contributing to reproductive dysfunction (67, 68). Conversely, adequate antioxidant intake has been shown to play a protective role in mitigating male infertility (61). Various dietary compounds, including polyphenols, carotenoids, and vitamins, contribute significantly to this effect by modulating mitochondrial function, maintaining the balance of reactive oxygen species, and enhancing mitochondrial biogenesis (62, 69–71).

In this study, the inclusion of hemp oil in the diet of Martina Franca jacks significantly influenced semen quality parameters and the lipid profiles of spermatozoa and erythrocyte membranes. These changes likely reflect the incorporation of PUFAs from hemp oil into cellular lipid matrices, a process essential for maintaining cellular function and membrane fluidity. Mammals, including equids, cannot synthesize PUFAs and must obtain them from dietary sources (72). Equine spermatozoa, like those of other mammals, contain high proportions of PUFAs, particularly DHA (omega-3) and docosapentaenoic acid [DPA, omega-6; (73)]. These findings indicate that hemp oil supplementation significantly impacts the lipid composition and oxidative stability of sperm membranes. Since PUFAs are critical for sperm function (74, 75), numerous studies have explored the potential of dietary interventions to enhance male fertility. Over the past decade, research has increasingly focused on using nutritional supplements to improve equine semen quality (76), with various nutraceuticals demonstrating potential benefits (77). The present study highlights the importance of nutraceuticals in optimizing sperm production and functionality in donkeys.

The addition of hemp oil had a pronounced effect on several semen quality parameters. The observed results align with previous on Martina Franca jacks conducted in Italy (47, 78). These studies, involving a similar number of animals and age range (7 animals, 4–10 years old), differed primarily in the number of ejaculates analyzed (35–364 ejaculates vs. 144 ejaculates in the present study). Over time, total and gel-free semen volumes decreased, possibly indicating a concentration effect in which volume is reduced while motility and morphology remains stable or improves. Specifically, motility and normal morphology percentages showed slight improvements, with a statistically significant increase in normal morphology, suggesting enhanced sperm health and viability. Few studies have investigated the influence of dietary n-3 PUFAs on equine reproductive performance, and none have specifically evaluated such supplementation in donkey diets. The reduction in semen concentration over time may suggest a trade-off between semen volume and sperm quality, where the nutritional benefits of hemp oil prioritize sperm viability over quantity. While several studies have reported beneficial effects of n-3 PUFA supplementation on sperm concentration in various species – including boars (79), rams (80), roosters (81), and humans (82, 83) – findings are inconsistent across different studies [e.g., (80, 84)]. Such discrepancies may be attributed to variations in dietary PUFA sources and levels, subject characteristics, or experimental conditions.

In this study, PUFA supplementation altered semen quality parameters as early as 15 days after the start of supplementation. The observed improvement in sperm motility is likely linked to modifications in sperm membrane composition induced by dietary PUFA supplementation (85). PUFAs enhance sperm flexibility, compressibility, deformability, and elasticity, ultimately improving membrane fluidity (74). This mechanistic pathway may explain the positive effects of dietary lipid supplementation on sperm motility. Similarly, the enhancement of sperm viability observed in this study is probably related to the antioxidant defense system of semen (86). The ability to counteract lipid peroxidation is crucial for maintaining sperm viability, as oxidative stress can compromise membrane integrity and cellular function (87). While no studies have specifically examined PUFA supplementation in donkeys, mixed results have been reported in horses. For example, Rodrigues et al. (76) found no changes in fresh semen quality after supplementing the diets of ten Mangalarga Marchador stallions with 150 mL of linseed-based PUFA oil. However, Brinsko et al. (88) and Elhordoy et al. (89) reported improved semen quality after cooling and post-thawing when DHA was added to stallions’ diets. The lipid composition of sperm membranes plays a crucial role in determining their functional properties. In this study, the observed improvement in semen quality may be attributed to modifications in the lipid profile of sperm plasma membranes. Previous research (62, 90) has highlighted DHA’s role in enhancing membrane resistance to osmotic stress, a key factor in maintaining sperm viability, particularly in cryopreservation contexts. PUFAs contribute to the structural integrity of sperm membranes by preserving the optimal fluidity required for fusion events during fertilization. According to Rodrigues et al. (76), PUFAs modulate the phase transition temperature of sperm membranes, delaying their transition from a liquid to a crystalline state. A higher PUFA content increases membrane flexibility, extending the duration in which cellular components remain in a liquid state and reducing the risk of ice crystal formation (91). This property enhances sperm resilience to sudden temperature fluctuations during freezing and thawing, ultimately improving post-thaw viability (76).

However, as noted by Van Tran et al. (92), the mechanisms of action of these molecules vary across species, leading to contrasting results. This variability poses challenges in interpreting the findings, particularly given the limited research on dietary additives’ effects on donkey semen quality. Studies in other species support the benefits of PUFA supplementation. For instance, Khoshvaght et al. (93) reported improved semen quality and sperm cryosurvival in Holstein bulls following supplementation with 3.5% fish oil. Similar findings have been reported in stallions (94) and Nili-Ravi buffalo bulls (95). These effects are often attributed to increased DHA content in sperm plasma membranes, which enhances sperm motility and morphology (96, 97). DHA and EPA are associated with enhanced membrane fluidity and sperm functionality (98). In particular, Khoshvaght et al. (93) proposed that increased incorporation of DHA into plasma membrane phospholipids, particularly in the sperm tail, may improve motility. This hypothesis is supported by Connor et al. (99), who reported that DHA is predominantly localized in the tail region of primate sperm. A high DHA content in the sperm tail appears to be critical for flagellar function and movement, both of which are essential for motility.

Conversely, an elevated ω6/ω3 ratio has been associated with increased oxidative stress and lipid peroxidation in sperm membranes. Excessive ω6 fatty acids, particularly arachidonic acid, contribute to reactive oxygen species production, negatively impacting sperm motility and viability. Adjusting dietary intake to favor ω3 fatty acids may mitigates oxidative damage and improve reproductive outcomes (100). In the present study, the observed decrease in SFAs and increase in monounsaturated fatty acids (MUFAs) suggest a shift toward greater membrane fluidity, which is consistent with improved sperm parameters. Similarly, lipidomic analysis of erythrocyte membranes revealed a comparable trends, with reduced SFAs and increased MUFAs contributing to enhanced membrane fluidity and deformability. Despite a slight reduction in ω3 PUFAs, the overall unsaturation index remained stable, indicating preserved membrane functionality. Additionally, the downward trend in the peroxidation index suggests reduced oxidative susceptibility, which may confer benefits to both erythrocyte and spermatozoa membranes. These findings align with previous studies emphasizing the pivotal role of dietary fatty acid composition in reproductive health (98).

## 5 Conclusion

This study provides novel insights into the effects of hemp oil supplementation on reproductive performance in Martina Franca jacks. The incorporation of omega-3 PUFAs into sperm and erythrocyte membranes enhances cellular functionality while reducing oxidative stress, ultimately contributing to improved sperm motility and morphology. These findings suggest that dietary hemp oil supplementation may serve as an effective strategy for enhancing reproductive efficiency in donkey breeding programs. Future research should investigate the long-term effects and optimal supplementation levels to support broader applications in equine and livestock reproduction.

## Data Availability

The raw data supporting the conclusions of this article will be made available by the authors, without undue reservation.
